# Resistance to imatinib in a *ETV6::PDGFRB* rearranged myeloid/lymphoid neoplasm with high-risk mutations: a case report

**DOI:** 10.3389/fonc.2025.1611747

**Published:** 2025-11-07

**Authors:** Lucia Cavelier, Leonie Saft, Birgitta Sander, Stefan Deneberg

**Affiliations:** 1 Department of Clinical Genetics and Genomics, Karolinska University Hospital, Solna, Sweden; 2 Department of Clinical Pathology and Cancer Diagnostics, Karolinska University Hospital and Institute, Stockholm, Sweden; 3 Center for Hematology and Regenerative Medicine, Department of Medicine Huddinge, Karolinska Institute, Karolinska University Hospital, Stockholm, Sweden

**Keywords:** platelet-derived growth factor receptor beta-rearranged myeloid/lymphoid neoplasms, treatment resistance, imatinib, case report, acute myeloid leukemia

## Abstract

Platelet-derived growth factor receptor beta (*PDGFRB*)-rearranged myeloid/lymphoid neoplasms (MLNs) are rare hematologic malignancies typically responsive to tyrosine kinase inhibitors (TKIs) such as imatinib. However, resistance—particularly in the context of co-occurring high-risk mutations—is uncommon and poorly characterized. We report a case of a 65-year-old man diagnosed with a *ETV6::PDGFRB*-translocated MLN, presenting as atypical chronic myeloid leukemia (aCML), who exhibited a brief response with development of resistance to imatinib. Although the patient initially achieved hematologic and partial cytogenetic remission, residual fibrosis and cytogenetic abnormalities persisted despite dose escalation. Molecular profiling revealed high-risk mutations in *ASXL1*, *KRAS*, *NRAS*, *SETBP1*, and *SRSF2*, along with a variant of uncertain significance (VUS) in *IDH2*. The patient progressed to acute myeloid leukemia (AML) within 11 months despite sequential therapies including dasatinib and azacitidine-venetoclax, ultimately succumbing to sepsis. This case highlights the limitations of TKI monotherapy in MLNs with *PDGFRB* rearrangements and co-existing high-risk mutations, underscoring the importance of early molecular profiling and consideration of allogeneic hematopoietic stem cell transplantation in cases with poor risk features.

## Introduction

Platelet-derived growth factor receptors alpha (PDGFRα) and beta (PDGFRß) are members of the class III receptor tyrosine kinase family, playing central roles in cellular growth, differentiation, and proliferation (1). Myeloid/lymphoid neoplasms with eosinophilia and *PDGFRB* rearrangements typically present as chronic myeloid neoplasms and are classified as a distinct entity in the World Health Organization (WHO) classification. The *ETV6* gene is the most common fusion partner (2).

These rearrangements result in constitutive tyrosine kinase activation, rendering most cases highly sensitive to imatinib (3, 4). Over 40 different fusion partners have been reported, with *ETV6* being the most frequent (5).

Patients with *PDGFRB* translocations may lack eosinophilia, only low grade in this case, and rarely present with additional cytogenetic or molecular abnormalities (4, 6–8).

Although most cases respond well to TKIs, resistance—either primary or acquired—remains poorly understood. Proposed mechanisms include secondary kinase domain mutations or disease progression to AML (9). TKI resistance has also been described in lymphoid malignancies with *PDGFRB* rearrangements (10, 11), though B-cell acute lymphatic leukemia with such fusions appears highly TKI-sensitive (12).

Here we describe a patient with a *ETV6::PDGFRB* translocation presenting as aCML, developing resistance to imatinib. The presence of high-risk mutations likely contributed to the resistance and rapid progression to AML. This case emphasizes the need for comprehensive molecular profiling, clonal monitoring, and early consideration of allogeneic stem cell transplantation in MLN cases with *PDGFRB* rearrangements that do not respond as expected to treatment.

## Case description

**Figure 1:**
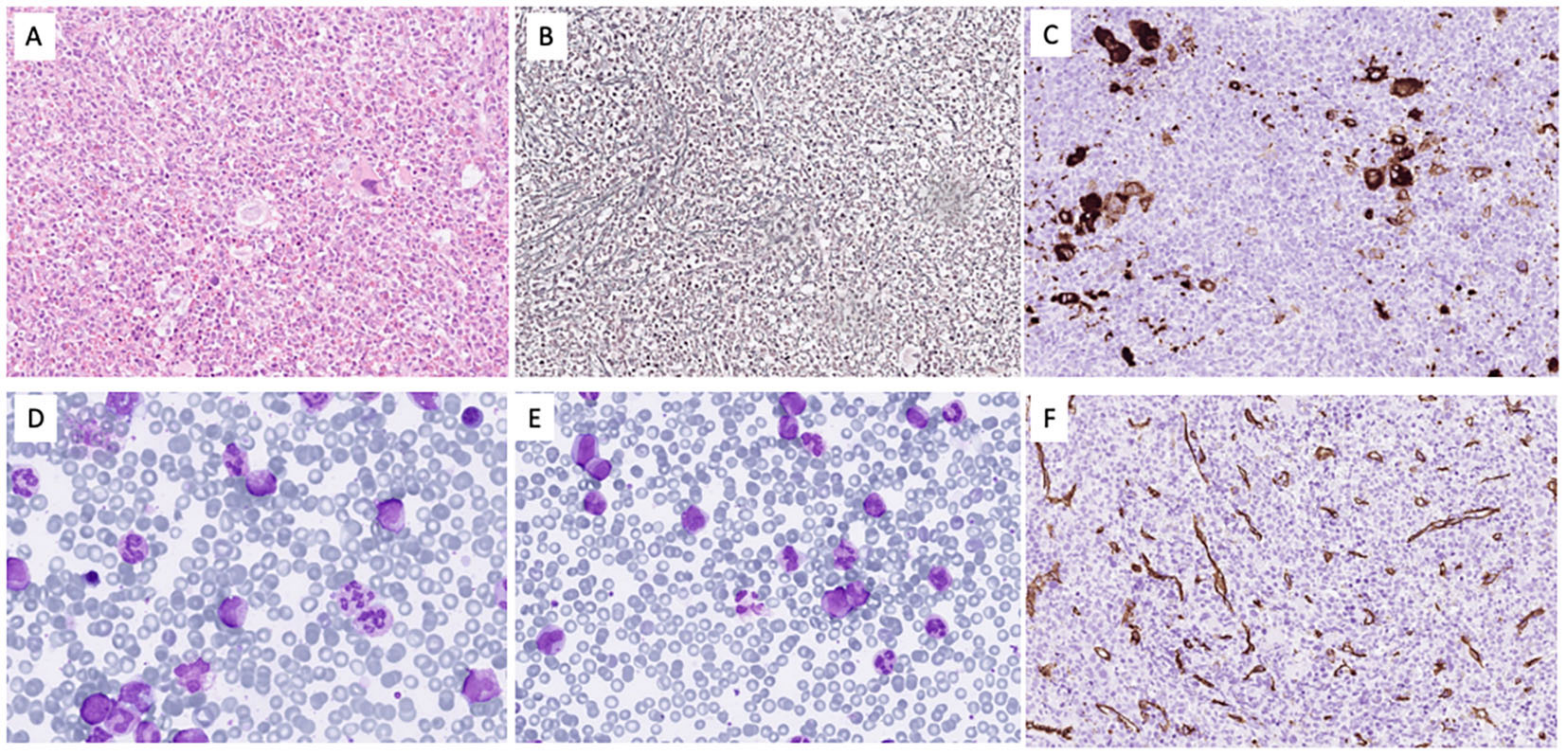
The bone marrow was hypercellular for age due to expansion of the granulocytopoiesis **(A)** with fibrosis grade II **(B)** and clusters of small megakaryocytes with monolobated nuclei **(C)**. In the bone marrow smears **(D)**, the M/E ratio was 8.3 with 1.8% blasts, 3% promyelocytes, and 22.6% myelocytes/metamyelocytes. The eosinophils were 1.6% and the monocytes 4%. In blood **(E)**, blasts constituted 1.5%, promyelocytes 3%, and myelocytes/metamyelocytes 14%. CD34 staining **(F)** showed no increase in blasts.

A 65-year-old man with a history of prostate cancer treated by radical prostatectomy 12 years prior, without adjuvant therapy, presented with splenomegaly (12 cm below the left costal margin), 8-kg weight loss over 2 months, and gout. Laboratory studies revealed leukocytosis (69 × 10^9^/L), mild peripheral eosinophilia (2%), and mild anemia. Bone marrow biopsy showed hypercellularity with myeloid proliferation, dysgranulopoiesis, and grade 2 fibrosis, without increased blasts or significant eosinophilia (**Figure 1**). Cytogenetics revealed t(5;12)(q33;p13) as the sole abnormality. Fluorescence *in situ* hybridization (FISH) examination confirmed *PDGFRB* rearrangement. RNA sequencing confirmed the presence of an *ETV6::PDGFRB* fusion transcript involving exon 4 of *ETV6* (NM_001987.5) and exon 9 of *PDGFRB* (NM_002609.4). Accordingly, whole genome sequencing identified the translocation breakpoint within intron 4 of *ETV6* (NM_001987.5) and intron 8 of *PDGFRB* (NM_001355017.2). *BCR::ABL1* was negative. Targeted sequencing revealed pathogenic mutations in *ASXL1*, *KRAS*, *NRAS*, *SETBP1*, *SRSF2*, and a VUS in *IDH2* (13).

Imatinib at 100 mg/day was initiated. Hematologic remission and spleen size normalization occurred within 2 months. However, at 10 months, a follow-up marrow showed only partial response, persistent patchy fibrosis, and 17% FISH positivity for the *PDGFRB* rearrangement. Imatinib was escalated to 400 mg/day, and transplantation workup was initiated.

Repeat marrow and FISH at 3 months later showed no further cytogenetic improvement and no blast increase. *PDGFRB* kinase domain sequencing showed no secondary mutations. Dasatinib at 100 mg/day was briefly trialed, but increasing leukocytosis necessitated azacitidine plus venetoclax. Despite therapy, the patient progressed to AML 15 months after diagnosis and died of sepsis with multiorgan failure during a neutropenic episode.

**Figure 2:**
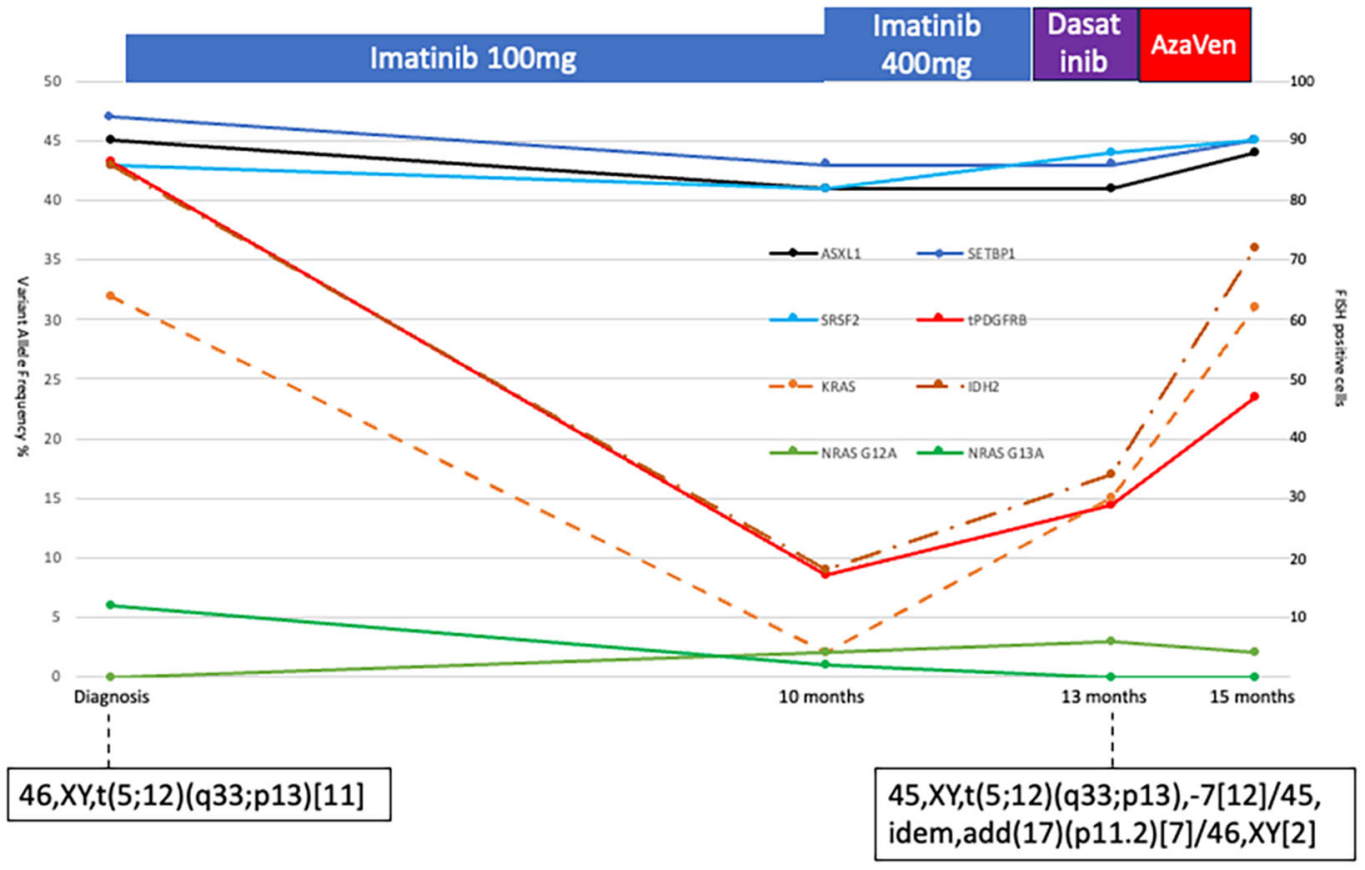
Diagram of bone marrow allele frequencies of *ASXL1*, *SETBP1*, *SRSF2*, *KRAS*, two *NRAS*, and IDH2 variants at different time points (scale on the left vertical). The *PDGFB*-translocation was measured with FISH at the same timepoints. A total of 200 cells were assessed, and the number of *PDGFB*-translocation-positive cells is plotted in the figure (red solid line, scale on the right vertical). Treatment is indicated above the diagram. Cytogenetic analyses were performed twice, and the results are indicated in the figure.

Serial sampling using the same targeted sequencing panel (13) in parallel with FISH for *PDGFRB*-rearrangement showed a decrease in *PDGFRB*-rearranged clone burden and parallel decreases in *KRAS/NRAS* variant allele frequency (VAF) (see **Figure 2**). In contrast, *ASXL1*, *SETBP1*, and *SRSF2* VAFs remained stable. Interestingly, the *IDH2* VUS VAF declined with the *PDGFRB* clone, suggesting that the variant was part of that clone. The two *NRAS* variants were consistently detected at low VAFs. Cytogenetics at AML transformation revealed monosomy 7 in 19/21 metaphases and a 17p11.2 locus loss in seven metaphases, alongside the persistent t(5;12), indicating clonal evolution. No *TP53* mutations or new molecular abnormalities were found (**Figure 2**).

## Discussion

Chronic-phase *PDGFRB*-rearranged neoplasms are typically sensitive to TKIs such as imatinib. Resistance is rare. Byrgazov et al. reported one imatinib-resistant *NDEL1::PDGFRB* case with a D850E mutation in the activation loop—a mechanism excluded in our case (9). Other reports describe TKI resistance predominantly in advanced-stage AML or lymphoid malignancies (7, 10, 11).

*PDGFRB* translocation alone appears to be a leukemogenic driver (14), explaining the usual absence of additional mutations or cytogenetic anomalies—similar to *BCR::ABL1* in CML. Among the rare cases of TKI failure, few underwent molecular profiling (15, 16), and no previously identified mutations have been clearly linked to resistance (7, 17).

An exception is a case by Gou et al., who described an *ETV6::PDGFRB*-positive patient with concurrent *NPM1*, *TET2*, and *NOTCH3* mutations and 46,XY,del(12)(p13p11.2), who developed a *PDGFR*-translocation positive T-lymphoblastic lymphoma while on imatinib (10).

In our case, *ASXL1*, *SETBP1*, and *SRSF2* VAFs remained unchanged during treatment, indicating their independence from the *PDGFRB*-positive clone. While *ASXL1* and *SRSF2* mutations may reflect age-related clonal hematopoiesis, *SETBP1* is more strongly associated with secondary AML, CMML, and aCML—often co-occurring with *ASXL1* and *SRSF2* in those neoplasms (18, 19). Given the unusual clinical course with an initial partial response to TKI followed by TKI-insensitive relapse and progression to AML, it is difficult not to think that the co-occurring mutations, in some way, contributed to the dismal outcome, even if it is impossible to conclude this in a single observational study such as this.

This case report has certain limitations, particularly in terms of generalizability—for example, the possibility of a pre-existing myeloproliferative neoplasm (MPN) with adverse mutations preceding the acquisition of the *PDGFRB* rearrangement cannot be excluded. Therefore, these findings should be interpreted with caution and may serve as a basis for further investigation.

The key message is to consider broad mutational profiling—even in the presence of targetable lesions like *PDGFRB* rearrangements and especially if signs of poor treatment response occur. In cases with poor molecular risk, rapid TKI response should not be assumed. While similar concerns have been raised in CML (20), this is, to our knowledge, the first report of primary TKI resistance in chronic-phase *PDGFRB*-rearranged MLN associated with high-risk mutations and adverse outcome.

## Data Availability

The raw data supporting the conclusions of this article will be made available by the authors, without undue reservation.
